# Beginning with the End-User in Mind: Application of Kern’s Six-Step Approach to Design and Create a Literary Journal for Healthcare Students

**DOI:** 10.15694/mep.2017.000054

**Published:** 2017-03-16

**Authors:** Adam Saperstein, Donovan Reed, Colin Smith, Brian Andrew

**Affiliations:** 1 Uniformed Services University of the Health Sciences; 2 San Antonio Military Medical Center; 3 Duke University Medical Center; 4 Naval Medical Center San Diego

**Keywords:** Literary Review, Kern, Poetry, Prose, Reflection

## Abstract

This article was migrated. The article was marked as recommended.

Expression through the arts has been shown to improve resilience and enhance patient care amongst healthcare trainees. This is all the more relevant when considering that many healthcare students feel that insitutions lack an outlet for articstic and creative expression. The creation of a student-run literary review is one possible strategy to allow learners to engage in artistic expression and mitigate rising burnout rates. Utilizing the Kern six-step model for curriculum development, we present a novel, replicable, and stepwise approach to designing a forum for artistic engagement in the form of a literary review.

## Introduction

Health professions students suffer from high levels of burnout, disillusionment, deterioration of communication skills, and decreased empathy, [ Cohen 2002; Dyrbye et al. 2008], which progressively worsen over time, and are associated with poorer patient care [ Shanafelt et al. 2002; Chen et al. 2016]. To help students cope with these stressors and enhance professional development, medical schools have sought to identify methods to improve resilience and enhance empathy [ Cooke et al. 2010]. By building resilience early in their professional education, providers may be able to incorporate wellness strategies during a time of dynamic professional identity formation and continue to build upon these strategies throughout the remainder of their careers.

Engagement with the arts, including reflective writing, poetry, prose, and visual art, may be particularly well suited to meet this need. Arts engagement enhances subject’s observation, communication, and collaboration skills, improves self-awareness, reduces burnout, and enhances empathy [ Reilly et al. 2005; Yang et al. 2011; Chen & Forbes 2014; Blease 2016]. In addition to these benefits, engagement with the arts provides an avenue for critical reflection and facilitates the development of leadership skills, professionalism and ethical behavior, each a critical component of provider competence [ Branson 2007; Cohen 2006]. At the Uniformed Services University (USU), an array of opportunities exists for students to engage with the arts, including open microphone events, expressive mask-making classes, humanities electives, medical improvisation seminars, and narrative mapping workshops. Positive feedback from learners prompted the following questions: (1) what unmet needs for arts engagement exist? (2) how can we increase learner engagement with the arts?

In this context, the Kern six-step model for curriculum development provided a framework for an innovative, replicable strategy to answer these questions. Using this model, we explored students’ views on the use of arts as a medium for professional development and enhanced empathy. We further identified barriers to student engagement with the arts, identified modalities ideally suited to overcome such barriers, and created a forum for arts engagement in the form of a student-driven, online and print literary review for federal healthcare students.

## Development Process

Our development process was guided by Kern’s six steps: [ Kern et al. 2009].

### (1) Problem Identification and General Needs Assessment

In spring 2015, we conducted a focus group of USU students and faculty, asking participants to comment on potential benefits of, and barriers to, opportunities for student engagement with the arts. In addition, participants were asked what, if any, artistic modalities they perceived to be of greatest value for their professional development and wellness.

Increased resilience and enhanced professional development were the most commonly cited potential benefits. The lack of a forum in which to share personal artistic expression and where learners could read, observe, and engage with the artistic expression of others was the most commonly identified limitation. The creation of a student-run literary review was identified as one possible strategy to overcome this limitation. This and other information gathered was used to develop a needs assessment for our targeted learners.

### (2) Needs assessment for targeted learners

In summer 2015, we sent a targeted needs assessment to 750 students via electronic survey. This assessment was designed to: (1) identify the degree to which the arts were incorporated into students’ curricula; (2) explore students’ views regarding use of arts as a medium for professional development; (3) identify barriers to engagement with the arts, and (4) explore whether students felt a healthcare student literary review was likely to overcome such barriers.

#### Results:

Among respondents (n = 121, response rate: 16%), 24.8% agreed or strongly agreed that literary or visual arts were included in their curricula. While nearly 55% reported that they had an outlet at their institution where they could express themselves artistically/creatively, fewer than 33% reported regularly engaging in any form of artistic expression.

Eighty-two percent of students reported being at least somewhat likely to read a student-run literary review, and greater than 50% agreed or strongly agreed that the presence of a literary and visual arts review would enhance professional development. Further, 67% agreed or strongly agreed that the presence of a literary arts review would contribute to the cultural identity of the federal healthcare student community.

Students preferred that the literary review be available in print and online formats, that the online version be delivered quarterly, and that it contain poetry, reflective writing, fiction and visual art.

Thereafter, we interviewed eight editors of diverse medical literary journals to identify best practices for creation of a literary review. Interviews were standardized and included questions regarding journal stratification and philosophy, editorial staff composition, roles, and workflow, journal content and formatting, and publication.

### (3) Goals and objectives

Based on needs assessment data, we established the goal of our literary review: To nurture and celebrate the finest art of federal healthcare students, to foster empathy and professional development by encouraging reflection on the human condition, and to cultivate a sense of community among federal healthcare students. Our objectives were: After reading the federal healthcare student literary journal, students, as demonstrated by comments offered during subsequent focus groups and/or survey responses will report: (1) an enhanced sense of community identity; (2) an enhanced sense of professional identity; and (3) enhanced empathy for their fellow human beings.

### (4) Educational Strategies

To achieve our goals, we endeavored to create a student-run literary review, available in print and online formats. An online presence was necessary to meet the stated needs of our users, to reach users who were not in close proximity to USU, and to help solicit content for future volumes. To prepare the student editorial team for its tasks, we organized a series of workshops taught by faculty advisors with the requisite expertise. Please refer to Table 1 for further description of the workshops.

### (5) Implementation

**Figure 1.**
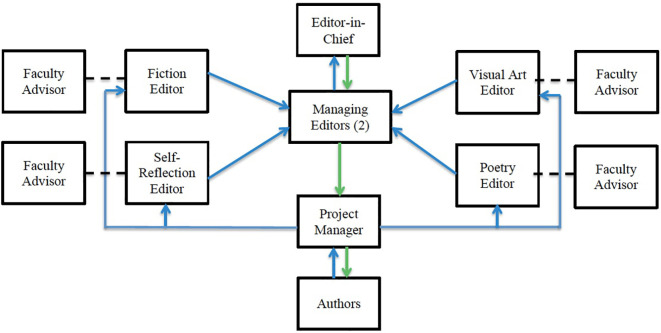
Healthcare Student Literary Review Organizational Chart. Submissions are received by project manager via email (blue arrow) and sent to appropriate student section editor for edits and recommendations. Managing editors then make additional edits and recommendations before sending pieces to Editor-in-chief, who makes third set of recommendations. Editor-in-chief sends submissions (green arrow) back to the managing editors, who together determine the final status of the piece. Project manager communicates all comments to author through email. During this process faculty advisors assist with any questions student may have.

Informed by survey results and editor interviews, we created an organizational structure for the literary review’s editorial staff. Please refer to Figure 1 for an overview of the organizational structure. Student editors were recruited for each role and standard operating procedures were established. Content was solicited using email, social media and personal networks. The review was primarily student-run, with faculty members available for guidance. Design and layout were completed by the editorial team, with the help of a professional graphic designer. In May 2016, the literary review, *Progress Notes*, was produced in print and online versions. ( https://goo.gl/FPKSwn)

#### Submissions:

We received 71 submissions: 28 visual art pieces, 12 reflections, 22 poems, and 9 fiction stories. Our final publication included 19 visual art pieces, 5 reflections, 11 poems, and 5 fiction stories, for an acceptance rate of 56%.

### (6) Evaluation and Feedback

The impact of this intervention on federal healthcare student empathy, resilience, and professional identity among healthcare students is yet to be determined, as the process of soliciting post-publication feedback is ongoing (see below).

## Discussion

Here we present a novel, stepwise approach to designing a forum for artistic engagement in healthcare trainees. Drawing from the curriculum development literature, we discovered that use of Kern’s Six-Step Model offered a blueprint for creation of a forum for arts engagement that is likely to be replicable at other institutions. We have created a model which (1) determines the art engagement needs of learners at a given institution (2) delineates arts engagement outcomes that are most likely to meet these needs (3) matches targeted outcomes to art-based strategies likely to achieve them (4) implements forums for arts engagement that are feasible and sustainable, and (with future projects) (5) evaluates the effectiveness of the implemented program, drawing on community member feedback to modify the forum to better meet those needs.

One barrier to broadly adopting this model may be the heavy demands placed on administrators and faculty at schools of healthcare professions, which could result in hesitancy to engage in the creation of activities perceived to make that burden even heavier. This may be especially true for activities more likely to be perceived as extracurricular. Our model addresses this concern as it is driven by students and is based on literature demonstrating the benefits of arts engagement [ Reilly et al. 2005; Branson 2007]. Further, despite recognition of the importance of the art and science of medicine, there remains a bias towards “hard sciences” that are perceived to be more “measurable” [ Cooke et al. 2010]. The inertia of this train of thought may impede creation of forums for arts engagement at some institutions. We feel that that use of the data driven Kern Model makes this more agreeable to those who would otherwise be hesitant to adopt an arts-based tool. Finally, given the unique nature of our sampled population, it is also reasonable to question the utility or desirability of a literary review as a generalizable intervention at other institutions. Instead we favor the broad use of this framework to create and adapt a locally desired product, fit to meet the needs of the local population.

In the future we aim to use qualitative and quantitative data to evaluate the extent to which our intervention resulted in students meeting our stated goals and objectives. We also hope to identify opportunities for improvements in helping students more effectively achieve these outcomes, and determine additional benefits that *Progress Notes* may offer students. Data obtained from these investigations will be analyzed and lessons learned will communicated to future leadership as part of continuous quality and process improvement in order to optimally meet the needs of our target population.

## Take Home Messages

• Engagement within the arts, including reflective writing, poetry, prose, and visual art may improve resilience and enhance empathy.
• Students within the health professions may feel their insitutions lack an outlet for articstic and creative expression.
• The creation of a student-run literary review is one possible strategy to allow learners to engage in artistic expression.
• The Kern six-step model provides an effective and practical framework for the development and implementation of a literary arts journal within the medical professions curriculum.

## Notes On Contributors

Adam Saperstein is an Associate Professor in the Department of Family Medicine at the Uniformed Services University School of Medicine and Daniel K. Inouye Graduate School of Nursing.

Donovan Reed is a graduate of the Uniformed Services University of the Health Sciences F. Edward Hebert School of Medicine. He is currently an Intern in General Surgery at the San Antonio Uniformed Services Health Education Consortium, where he has been selected for residency in Ophthalmology.

Colin Smith is a graduate of the Uniformed Services University of the Health Sciences F. Edward Hebert School of Medicine. He is currently an Internal Medicine/Psychiatry resident at Duke University.

Brian Andrew is a graduate of the Uniformed Services University of the Health Sciences F. Edward Hebert School of Medicine. He is currently an Internal Medicine Intern at Naval Medical Center, San Diego.
