# Supplementary material for: Beginning with the End-User in Mind: Application of Kern’s Six-Step Approach to Design and Create a Literary Journal for Healthcare Students
Source: MedEdPublish (2016). 2017 Mar 16;6:54. [Version 1] doi: 10.15694/mep.2017.000054 (PMC10885245; doi:10.15694/mep.2017.000054)
Supplement: Peer review history

# Reviewer report: Reviewer response for version 1

**DOI:** 10.21956/mep.19461.r28689

**Published:** 2019-03-31

**Authors:** Ken Masters

**Affiliations:** 1 Sultan Qaboos University

This review has been migrated. The reviewer awarded 4 stars out of 5 A very nicely-structured and written paper exploring the processes leading to the introduction of a literary review for medical students, as a method of allowing them artistic expression and reflection. The authors have followed a very structured approach to their implementation, gathering information from the students and from external experts (editors and faculty advisors) in order to ensure that they meet the needs of the students and also meet expected standards and best practice.From a brief look at the journal, I would say that it appears to be going strong. It would be valuable for the authors to write a follow-up article on what has been learnt and what has changed in the journal since that first issue.One note: for interested readers, the authors should also have supplied the URL for the journal (https://www.usuhs.edu/students/progress-notes-literary-review) rather than only to the first issue.

Reviewer Expertise:

NA

No decision status is available

# Reviewer report: Reviewer response for version 1

**DOI:** 10.21956/mep.19461.r28688

**Published:** 2017-03-19

**Authors:** Subha Ramani

**Affiliations:** 1 Harvard Medical School, Brigham and Women's Hospital

This review has been migrated. The reviewer awarded 5 stars out of 5 Teaching humanism, resilience and other essential skills in the era of multiple commitments and shrinking time, certainly requires inclusion of artistic and creative expressions in curricula. Burn-out is of increasing concern in the medical profession and teaching trainees and faculty resilience is one remedy to promote wellness. I agree with the authors that incorporation of art not only promotes empathy / compassion among healthcare professionals, but also enhances their observation skills which are required clinical skills. What may be novel in this study is that the students lead the curriculum, and it is modelled on a well-known and established curricular framework from the Johns Hopkins group. The paper is well written, well referenced with an appropriate problem statement, and educational framework. It also emphasises the learning outcomes and the importance of program evaluation and based on needs assessment- general and targeted. One limitation is the low response rate in the needs assessment phase, so it is hard to extrapolate the usefulness of this area to all students, especially the non-respondents. I do hope the authors will continue to the next steps of program evaluation as they have indicated. For this subject, sound qualitative outcomes would be very important.I believe this paper would be useful to all those involved in health professions education, regardless of their speciality. Even those not interested in designing a similar curriculum could use these steps in designing other curricula,

Reviewer Expertise:

NA

No decision status is available
